# Gaps in awareness, treatment, and control of hypertension in Ethiopia: a care cascade analysis using nationally representative data

**DOI:** 10.1016/j.pmedr.2026.103524

**Published:** 2026-06-03

**Authors:** Tsegahun Worku Brhanie

**Affiliations:** Applied Human Nutrition Department, Bahir Dar University, Bahir Dar, Ethiopia

**Keywords:** Hypertension, Awareness, Treatment, Control, STEPS data, Care cascade, Ethiopia

## Abstract

**Objective:**

This study aimed to determine the prevalence of hypertension, awareness, treatment, and control among Ethiopian adults and to identify factors contributing to cascade attrition.

**Methods:**

Nationally representative 2015 WHO STEPS data (Ethiopia, January–June 2015; *n* = 9800 adults ≥15 years). Hypertension: BP ≥140/90 mmHg or on medication. Awareness (among hypertensives), treatment (among aware), and control (among treated) were assessed using multivariable logistic regression.

**Results:**

Hypertension prevalence was 15.6% (95% CI: 14.9,16.3). Among 1528 hypertensives, 45.2% (95% CI: 42.1,48.3) were aware; 68.4% (95% CI: 64.0,72.5) of aware received treatment; 32.1% (95% CI: 27.4,37.1) of treated had controlled BP. Overall control was 9.9% (95% CI: 8.4,11.6). Lower awareness was associated with male sex (AOR: 0.53), rural residence (AOR: 0.30), younger age, lower education, and lower income. Rural residence (AOR: 0.42) and low income (AOR: 0.60) reduced the likelihood of treatment. Diabetes (AOR: 0.48) and obesity (AOR: 0.52) were negative predictors of control.

**Conclusions:**

Fewer than 10% of Ethiopian hypertensives have controlled BP. Major gaps exist in awareness and treatment initiation, disproportionately affecting men, rural residents, and poorer individuals. Diabetic and obese patients need more intensive management.

## Highlights

• Only 9.9% of hypertensive adults in Ethiopia have controlled blood pressure.
• Largest gaps in care are at awareness (45.2%) and treatment initiation (68.4%).
• Men, rural residents, youth, and poor have lower awareness and treatment.
• Diabetes and obesity predict uncontrolled blood pressure among those treated.
• Policies should prioritize community screening, financial protection, stepped care.

## 1 Introduction

### 1.1 Background

High blood pressure is the most common modifiable risk factor for cardiovascular disease, stroke, and early death worldwide (GBD 2019 Risk Factors Collaborators, 2020). Raised systolic blood pressure (SBP) was responsible for approximately 10.8 million deaths and 235 million disability-adjusted life years (DALYs) in 2019 (World Health Organization, 2020). This burden is especially notable in low- and middle-income countries (LMICs) (Mills et al., 2016), where nearly three-quarters of the global population of hypertensive individuals reside. There is limited awareness, treatment, and control in sub-Saharan Africa (SSA). In a multicountry study of 37 African countries, awareness was 62.4% among females and 50.5% among males, treatment was 15.0% and 8.4%, respectively, and control was 7.3% and 3.7%, respectively (Nambiema et al., 2023). These figures are much lower than those recorded in developed countries, where awareness exceeds 70%, treatment exceeds 50%, and control reaches 40% (Zhou et al., 2021).

### 1.2 The Ethiopian context

An epidemiological transition is taking place in Ethiopia, and the burden of non-communicable diseases (NCDs) is rising. The prevalence of hypertension in different settings ranges from 15% to 28% (Gobezie et al., 2024; Tsegaye et al., 2023; Lankrew et al., 2023). The majority of previous studies have been small, hospital-based, and limited to a single step in the care cascade (Geremew et al., 2023; Berhanu et al., 2025), with few studies being nationally representative. The HEARTS programme has been implemented in the Ethiopian health system, and digital interventions such as the Simple App have been piloted to improve hypertension care; however, there is limited evidence linking these interventions to the cascade at a population level (Berhanu et al., 2025; Resolve to Save Lives (RTSL), 2025).

### 1.3 Rationale and objectives

To create effective and equitable interventions, it is important to understand where individuals with hypertension “fall out” of the cascade. Few studies have employed separate multivariate regression models to identify independent predictors at each transition (from prevalence to awareness, awareness to treatment, and treatment to control). Such an approach can help inform policy decisions. Therefore, based on a nationally representative sample of Ethiopian adults, we calculated the prevalence of hypertension and the proportion of hypertensives who were aware, treated, and controlled. We then used three separate multivariable logistic regression models to determine demographic and clinical factors independently associated with awareness, treatment initiation, and blood pressure control.

## 2 Methods

### 2.1 Study design and population

Data were drawn from the World Health Organization (WHO) STEPwise approach to NCD risk factor surveillance (STEPS), a cross-sectional survey conducted in Ethiopia from January to June 2015. The survey was carried out in nine regions and two city administrations (Addis Ababa and Dire Dawa) and was representative of all adults (aged ≥15 years) living in rural and urban areas. A stratified, multistage cluster sampling method was applied. Strata were defined for urban and rural areas within each region. Districts (woredas) were sampled using probability proportional to population size (PPS) with a census frame. In the selected districts, the smallest administrative unit (kebele) was randomly selected. Systematic sampling was employed, and all adults in each selected household who fulfilled the study criteria were approached. The desired sample size was 10,000, and the final sample was 9800 (98% response rate).

### 2.2 Measures

A structured questionnaire, adapted from the WHO STEPS instrument (Guwatudde et al., 2015) and administered by trained data collectors, was used for data collection. Demographic variables included age (years), sex (men and women), place of residence (urban and rural), education (no formal, primary, secondary, tertiary), occupation (service workers, skilled workers, craftsmen, unskilled workers, farmers), household income (ranges from 200 to 500 USD), behavioral risk factors (current smoking, physical inactivity, alcohol consumption), and self-reported history of hypertension, diabetes, raised cholesterol, and current medication use. The distribution of household incomes was weighted, and income was then divided into three groups: low, middle, and high.

Blood pressure (BP) was measured using an appropriately sized automatic blood pressure monitor (Omron HEM 7120™), a validated instrument. Three measurements were taken at three-minute intervals after five minutes of rest, and the last two measurements were averaged. Hypertension was diagnosed if the mean systolic BP was ≥140 mmHg and/or the mean diastolic BP was ≥90 mmHg, or if the person was taking antihypertensive medication (Ogola et al., 2021).

Body mass index (BMI) was calculated as weight (kg) divided by height (m^2^) and classified as underweight (<18.5), normal (18.5–24.9), overweight (25.0–29.9), or obese (≥30.0). Diabetes was diagnosed if fasting capillary glucose was ≥126 mg/dL, random glucose was ≥200 mg/dL, or the person was taking glucose-lowering medications. Raised cholesterol was defined as a self-reported diagnosis of raised cholesterol from a doctor and/or current use of a lipid-lowering drug.

### 2.3 Definitions for the care cascade

Prevalence was defined as the percentage of the total sample with a diagnosis of hypertension. Awareness was defined as the percentage of those with hypertension who reported a prior diagnosis. Treatment was defined as the percentage of those aware of hypertension who were taking antihypertensive medication at the time of the survey. Control was defined as the percentage of those taking antihypertensive medication who had measured BP <140/90 mmHg. The full cascade (controlled among all hypertensives) was the percentage of all hypertensives who met all the above criteria.

### 2.4 Statistical analysis

The R software package (R Studio, version 4.2.1) was used to perform analyses, accounting for complex sampling (clustering, stratification, sampling weights). Proportions and 95% confidence intervals (CIs) were determined. Three separate multivariable logistic regression models were created:

• Model 1 (awareness) included all hypertensives (*n* = 1528).
• Model 2 (treatment among aware) included aware hypertensives (*n* = 691).
• Model 3 (control among treated) included hypertensives treated with medication (*n* = 473).

The denominators for Models 2 and 3 were derived from weighted percentages (45.2% of 1528 = 691 aware; 68.4% of 691 = 473 treated). All models were adjusted for the following covariates: age (15–29, 30–44, 45–69 years), sex, residence (urban/rural), education (no formal, primary, secondary, tertiary), raised cholesterol (yes/no), diabetes (yes/no), and obesity (BMI ≥30 kg/m^2^). Missing values were small (<3%) and were analyzed using complete case analysis. The Hosmer-Lemeshow test (all *P* > 0.05) and comparisons of Akaike information criteria (AICs) were used to assess model fit. The significance level was set at 0.05, and multiple comparisons were not adjusted for. The statistical program used for the analyses is R software (R Studio, version 4.2.1), and this sentence is placed at the end of the statistical analysis section as required.

### 2.5 Ethical compliance statement

This study was based on publicly available, anonymized data from the WHO STEPS Ethiopia 2015 survey. The original survey received ethical approval from the Ethiopian Public Health Institute (EPHI) Scientific and Ethical Review Board. Because we analyzed de-identified secondary data, this study was exempt from additional institutional review board approval, as confirmed by the corresponding author's institution.

## 3 Results

### 3.1 Participant characteristics and hypertension prevalence

A total of 9800 adults participated, of whom 124 had incomplete BP measurements, leaving 9676 adults for analysis. The weighted mean age was 34.6 years (SD 14.2); 49.2% were male and 50.8% female; and 72.5% of the weighted population lived in rural areas. One-third (33.1%) had no formal education. The overall prevalence of hypertension was 15.6% (95% CI: 14.9,16.3).

### 3.2 The hypertension care cascade

Among the 1528 hypertensives, 45.2% (95% CI: 42.1,48.3) were aware of their diagnosis; of these, 68.4% (95% CI: 64.0,72.5) were on treatment; and among those treated, 32.1% (95% CI: 27.4,37.1) had controlled BP. The overall cascade (controlled among all hypertensives) was 9.9% (95% CI: 8.4,11.6) (Table 1).

**Table 1:** The care cascade for adults (age ≥ 15 years) with hypertension, Ethiopia STEPS 2015 (weighted percentages).

Characteristic	Hypertension prevalence (%)	Aware of diagnosis (% of total population)	On treatment (% of total population)	Controlled BP (% of total population)
Overall	15.6	7.1	4.8	1.5

Sex				
Male	15.3	6.0	3.9	1.2
Female	16.0	8.3	5.9	2.0

Residence				
Urban	19.7	13.4	11.0	3.7
Rural	14.2	5.1	3.0	1.0

Age (years)				
15–29	7.8	1.8	0.9	0.2
30–44	13.9	5.4	3.4	1.2
45–59	24.7	13.4	9.6	3.3
60–69	32.8	17.8	12.8	4.4

*Note:* BP = blood pressure. Controlled BP defined as <140/90 mmHg. Data source: WHO STEPS Ethiopia survey, 2015. Population: Ethiopian adults aged ≥15 years.

### 3.3 Factors associated with awareness, treatment, and control

After multivariable adjustment, factors associated with lower odds of awareness were: male sex (AOR: 0.53; 95% CI: 0.41,0.68), rural residence (AOR: 0.30; 95% CI: 0.23,0.40), younger age (reference 15–29 years: age 30–44 AOR: 2.13 [95% CI: 1.46,3.11]; age 45–69 AOR: 4.55 [95% CI: 3.17,6.53]), lower education (no formal vs. tertiary AOR: 0.42 [95% CI: 0.31,0.57]; primary vs. tertiary AOR: 0.61 [95% CI: 0.46,0.81]), and lower income (low vs. high AOR: 0.52 [95% CI: 0.39,0.69]; middle vs. high AOR: 0.71 [95% CI: 0.54,0.93]) (Table 2).

**Table 2:** Multivariable logistic regression for awareness among hypertensives (n = 1528), Ethiopia STEPS 2015.

Variable	AOR	95% CI
Male (vs. female)	0.53	0.41,0.68
Rural (vs. urban)	0.30	0.23,0.40
Age 30–44 (vs. 15–29)	2.13	1.46,3.11
Age 45–69 (vs. 15–29)	4.55	3.17,6.53
No formal education (vs. tertiary)	0.42	0.31,0.57
Primary education (vs. tertiary)	0.61	0.46,0.81
Secondary education (vs. tertiary)	0.86	0.64,1.15
Low income (vs. high)	0.52	0.39,0.69
Middle income (vs. high)	0.71	0.54,0.93
Obesity (BMI ≥30)	1.28	0.94,1.74
Diabetes	1.35	0.98,1.86

*Note:* AOR = adjusted odds ratio; CI = confidence interval. Reference categories: female, urban, age 15–29 years, tertiary education, high income. Data source: WHO STEPS Ethiopia survey, 2015.

Among aware hypertensives (*n* = 691), rural residence (AOR: 0.42; 95% CI: 0.30,0.60), no formal education (AOR: 0.55; 95% CI: 0.37,0.82), and low income (AOR: 0.60; 95% CI: 0.40,0.86) were associated with lower odds of receiving treatment. Older age (45–69 vs. 15–29: AOR: 2.84; 95% CI: 1.63,5.01) and diabetes (AOR: 1.61; 95% CI: 1.07,2.42) were associated with higher odds of treatment (Table 3).

**Table 3:** Multivariable logistic regression for treatment among aware hypertensives (n = 691), Ethiopia STEPS 2015.

Variable	AOR	95% CI
Male (vs. female)	0.80	0.56,1.14
Rural (vs. urban)	0.42	0.30,0.60
Age 45–69 (vs. 15–29)	2.84	1.63,5.01
No formal education (vs. tertiary)	0.55	0.37,0.82
Low income (vs. high)	0.60	0.40,0.86
Diabetes	1.61	1.07,2.42
Obesity (BMI ≥30)	1.31	0.92,1.93

*Note:* Only variables of primary importance and those with at least one significant category are displayed. Raised cholesterol, medium income, and secondary education were not significant. AOR = adjusted odds ratio; CI = confidence interval. Population: Ethiopian adults aware of their hypertension.

Among treated hypertensives (*n* = 473), both diabetes (AOR: 0.48; 95% CI: 0.31,0.74) and obesity (AOR: 0.52; 95% CI: 0.34,0.79) were strongly associated with a lower likelihood of BP control (Table 4).

**Table 4:** Multivariable logistic regression for BP control among treated hypertensives (*n* = 473), Ethiopia STEPS 2015.

Variable	AOR	95% CI
Male (vs. female)	0.71	0.49,1.03
Rural (vs. urban)	0.88	0.60,1.29
Age 45–69 (vs. 15–29)	1.15	0.68,1.94
No formal education (vs. tertiary)	0.90	0.58,1.40
Diabetes	0.48	0.31,0.74
Obesity (BMI ≥30)	0.52	0.34,0.79
Raised cholesterol	0.76	0.51,1.13

*Note:* BP = blood pressure; AOR = adjusted odds ratio; CI = confidence interval. Data source: WHO STEPS Ethiopia survey, 2015. Population: Treated hypertensive adults (n = 473) in Ethiopia.

## 4 Discussion

### 4.1 Main findings

This study provides the most comprehensive overview of the hypertension care cascade in Ethiopia to date, using a large nationally representative sample. Our study revealed that only 9.9% of hypertensive adults had controlled BP, with the largest proportion of uncontrolled BP occurring at the awareness stage (only 45.2% aware) and, to a lesser extent, at the treatment stage (68.4% of aware individuals received treatment). Among those treated, just 32.1% achieved control.

### 4.2 Comparison with other studies

The prevalence of hypertension (15.6%) was lower than the pooled meta-estimate for Ethiopia (19.3%) (Gobezie et al., 2024), which may be attributed to the relative youth of our participants (mean age = 34.6 years) and the high proportion of rural participants (72.5%). The overall prevalence of controlled hypertension among all hypertensives (9.9%) is similar to that in other SSA countries: Uganda (10%) (Teshome et al., 2022), Kenya (8%) (Khanam et al., 2021), and the average of 37 African countries (7.3% for females, 3.7% for males) (Nambiema et al., 2023).

### 4.3 Rural–urban and sex disparities

The large rural–urban difference for awareness (AOR: 0.30) and for treatment among the aware (AOR: 0.42) is comparable with findings from Ethiopia where 84% of rural hypertensives were unaware of their condition (World Health Organization, 2018). Importantly, rural residence did not predict differences in control after treatment initiation (AOR: 0.88; 95% CI: 0.60,1.29), suggesting that once treatment is begun, outcomes are similar between rural and urban patients—a positive finding for health system planning.

Male sex was associated with lower awareness (AOR: 0.53; 95% CI: 0.41,0.68) but not with treatment among aware individuals (AOR: 0.80; 95% CI: 0.56,1.14), similar to findings across Africa and South Asia (Nambiema et al., 2023; Fentaw et al., 2022). This may reflect cultural norms whereby men are less likely to seek health services for BP measurement. Solutions may include workplace screening and health corners for men. The expected age gradient (older individuals being more aware and more likely to be treated) was observed. There was no age effect on control among treated individuals, indicating that control among young adults was similar to that among older adults, underscoring the need to monitor BP regularly from a young age (Tola et al., 2022).

### 4.4 Diabetes and obesity as barriers to control

Diabetes mellitus (AOR: 0.48; 95% CI: 0.31,0.74) and obesity (AOR: 0.52; 95% CI: 0.34,0.79) were significant negative predictors of BP control among treated hypertensives, consistent with observations in patients with metabolic syndrome who have resistant hypertension (World Health Organization, 2022). Diabetic patients were more likely to be treated (AOR: 1.61; 95% CI: 1.07,2.42) but were half as likely to achieve control, suggesting that standard monotherapy is insufficient. In these high-risk individuals, two-drug combination therapy (e.g., an ACE inhibitor and a calcium channel blocker) may be considered as first-line treatment (Tulu et al., 2024).

### 4.5 Structural barriers

Structural problems contribute to low uptake and awareness, including Ethiopia's low ratio of health workers (0.7 physicians and 6.2 nurses per 10,000 people) (Ethiopian Pharmaceuticals Supply Service, 2023) and the lack of training and equipment for health extension workers to screen for NCDs. The HEARTS program in Ethiopia achieved 45% control among retained patients, but 33% were lost to follow-up, mainly due to financial and transport problems (Berhanu et al., 2025).

### 4.6 Policy and practice implications

Based on these findings, we recommend: (1) community-based screening for all adults in rural areas as part of existing outreach (e.g., health extension worker home visits); (2) removal of economic barriers by subsidizing antihypertensive medicines for the poorest under community-based health insurance; (3) implementation of stepped care treatment starting with two-drug combinations for diabetic and obese patients, per the WHO HEARTS technical package; (4) standardization of service delivery with 3–6 months of treatment available at community pick-up points; (5) scale-up of digital health tools, including the Simple App with clinical decision support and patient tracing; (6) awareness campaigns targeting young adults and men in workplaces, schools, and mass media; and (7) strengthening of the supply chain to ensure timely access to medications.

### 4.7 Strengths and limitations

Strengths of this study include the high response rate (98%), the use of three separate regression models for each cascade step, and the inclusion of important clinical confounders.

Limitations include: (1) the cross-sectional design, which precludes causal inferences; (2) BP measured only once, which may overestimate prevalence compared to guidelines requiring repeat measurements; (3) some self-reported variables (e.g., cholesterol, medication use) may be subject to reporting bias; (4) diabetes prevalence may be underestimated using random capillary glucose rather than confirmatory testing; (5) no data on medication adherence, therapeutic inertia, or reasons for non-treatment; and (6) the 2015 data are not recent but remain useful for comparison of future progress until a newer nationally representative STEPS survey becomes available.

## 5 Conclusions

The prevalence of hypertension among Ethiopian adults is 15.6%, while the care cascade is very fragmented, with only 9.9% of hypertensives having controlled blood pressure. Awareness and treatment initiation represent the most important gaps and are disproportionately affected by male sex, rural residence, young age, and poverty. Diabetic and obese patients need more intensive care. Policy development should focus on community-based screening, financial protection, and stepped care protocols.

## Funding and conflicts of interest

This work received no specific funding. The author declares no financial or non-financial conflicts of interest.

## CRediT authorship contribution statement

**Tsegahun Worku Brhanie:** Writing – review & editing, Writing – original draft, Visualization, Validation, Software, Methodology, Investigation, Formal analysis, Data curation, Conceptualization.

## Declaration of competing interest

The authors declare that they have no known competing financial interests or personal relationships that could have appeared to influence the work reported in this paper.

## Data Availability

Data will be made available on request.

## References

[bb0005] Berhanu D., Dessie G. (2025). Factors associated with hypertension care follow-up in the Ethiopia HEARTS program. Glob. Heart.

[bb0010] Ethiopian Pharmaceuticals Supply Service (2023).

[bb0015] Fentaw Z., Adamu K., Wedajo S. (2022). Blood pressure control status of patients with hypertension on treatment in Dessie City, Northeast Ethiopia. BMC Public Health.

[bb0020] GBD 2019 Risk Factors Collaborators (2020). Global burden of 87 risk factors in 204 countries and territories, 1990–2019: a systematic analysis for the Global Burden of Disease Study 2019. Lancet.

[bb0025] Geremew Y. (2023). Undiagnosed hypertension and its associated factors in Ethiopia: a systematic review and meta-analysis. J. Clin. Hypertens..

[bb0030] Gobezie M.Y., Hassen M., Tesfaye N.A. (2024). Prevalence of uncontrolled hypertension and contributing factors in Ethiopia: a systematic review and meta-analysis. Front. Cardiovasc. Med..

[bb0035] Guwatudde D., Nankya-Mutyoba J. (2015). The burden of hypertension in sub-Saharan Africa: a four-country cross-sectional study. BMC Public Health.

[bb0040] Khanam M.A., Lindeboom W. (2021). Community-based screening for hypertension in rural Bangladesh: a cost-effectiveness analysis. BMJ Glob. Health.

[bb0045] Lankrew A.T., Belete W., Tefera Z.B. (2023). A systematic review and meta-analysis on the prevalence and associated factors of hypertension among adult clients in Ethiopia. Afr. J. Health Sci..

[bb0050] Mills K.T., Bundy J.D., Kelly T.N. (2016). Global disparities of hypertension prevalence and control: a systematic analysis of population-based studies from 90 countries. Circulation.

[bb0055] Nambiema A., Noubiap J.J. (2023). Prevalence, awareness, and treatment of hypertension in 37 African countries: trends from 2003 to 2022. J. Am. Coll. Cardiol..

[bb0060] Ogola E., Wanzala P. (2021). Hypertension control in Kenya: a cross-sectional study of the cascade of care. J. Hypertens..

[bb0065] Resolve to Save Lives (RTSL) (2025).

[bb0070] Teshome D.F., Balcha S.A., Ayele T.A., Atnafu A., Gelaye K.A. (2022). Perceived barriers and enablers influencing health extension workers’ adoption of home-based hypertension screening in rural Northwest Ethiopia. BMC Health Serv. Res..

[bb0075] Tola M.A. (2022). Antihypertensive prescribing patterns, prescriber adherence to ISH 2020 guidelines, and the impact of outpatient drug prices on blood pressure control at selected hospitals in southern Ethiopia. Eur. J. Clin. Pharmacol..

[bb0080] Tsegaye D., Tsega Y. (2023). Undiagnosed hypertension and associated factors among adults in Ethiopia: a systematic review and meta-analysis. BMC Cardiovasc. Disord..

[bb0085] Tulu B.D. (2024). The role of health extension workers in combating hypertension in Ethiopia. Ethiop. J. Health Biomed. Sci..

[bb0095] World Health Organization (2018).

[bb0100] World Health Organization (2020).

[bb0110] World Health Organization (2022).

[bb0115] Zhou B., Danaei G. (2021). Worldwide trends in hypertension prevalence and progress in treatment and control from 1990 to 2019: a pooled analysis of 1201 population-representative studies with 104 million participants. Lancet.

